# Systematic review of azacitidine regimens in myelodysplastic syndrome and acute myeloid leukemia

**DOI:** 10.1186/s12878-017-0094-8

**Published:** 2018-01-31

**Authors:** Roman M. Shapiro, Alejandro Lazo-Langner

**Affiliations:** 1 Department of Medicine, Western University, London, ON Canada; 2 Department of Medicine, Division of Hematology, Western University, London, ON Canada; 3 Department of Epidemiology & Biostatistics, Western University, London, ON Canada; 4 Hematology Division, London Health Sciences Centre, 800 Commissioners Rd E, Rm E6-216A, London, ON N6A 5W9 Canada

**Keywords:** Azacitidine, Dosing, Myelodysplastic, Leukemia

## Abstract

**Background:**

5-Azacitidine administered as a 7-day dosing regimen (7–0-0) is approved in high risk IPSS myelodysplastic syndrome (MDS) patients. Alternative regimens such as a 5-day (5–0-0) or 7-day with a weekend break (5–2-2) are commonly used. No randomized controlled trial has been done directly comparing all three dosing regimens. The objective of this study was to compare the efficacies of the 5–0-0, 5–2-2, and 7–0-0 regimens in MDS and AML.

**Methods:**

A systematic review was conducted using MEDLINE, EMBASE and CENTRAL. Eligible studies were randomized controlled trials (RCTs), observational prospective and retrospective studies. The primary clinical outcomes were Objective Response Rate (ORR) defined as the sum of complete response (CR), partial response (PR), and hematological improvement (HI) as defined by the IWG 2006 criteria. A meta-analysis of simple proportions was conducted using a random effects model with weights defined according to Laird and Mosteller. Comparisons between groups were not attempted due to the heterogeneity of study designs.

**Results:**

The only RCT directly comparing alternative azacitidine regimens showed no difference in ORR between the 5–0-0 and 5–2-2 regimens. All other RCTs compared a dosing regimen to conventional care. The pooled proportion of ORR was 44.8% with 95% CI (42.8%, 45.5%) for 7–0-0, 41.2% with 95% CI (39.2%, 41.9%) for 5–0-0, and 45.8% with 95% CI (42.6%, 46.4%) for 5–2-2.

**Conclusions:**

Indirect comparison of alternative azacitidine dosing regimens in MDS and AML shows a benefit for the 7-day regimen in attaining ORR. Additional RCTs are required to definitively address this comparison.

**Electronic supplementary material:**

The online version of this article (10.1186/s12878-017-0094-8) contains supplementary material, which is available to authorized users.

## Background

Azacitidine has become the standard of care for patients with high risk myelodysplastic syndrome (MDS) when a hematopoietic stem cell transplant is not an option. In the CALGB 9221 randomized clinical trial, azacitidine administered at 75 mg/m^2^ for 7 continuous days resulted in an objective response rate of 16% compared to no response in the control group [1, 2]. This response rate included improvement in peripheral cytopenias resulting in transfusion independence as well as a reduction in the bone marrow blast percentage [2]. The subsequent international phase III open label randomized controlled trial (RCT) AZA-001 comparing azacitidine to conventional care that included low dose cytarabine, best supportive care or intensive chemotherapy showed a statistically significant survival benefit as well as a doubling in the time to progression to AML with azacitidine. The results of the AZA-001 clinical trial led to the FDA extending a survival benefit to the use of the drug in intermediate-2/high risk MDS by international prognostic scoring criteria (IPSS), CMML with 10–30% blasts, and AML with 20–30% blasts [3].

The standard approved dose cycle of azacitidine has been 75 mg/m^2^ for 7 continuous days (7–0-0), according to the AZA-001 and CALGB clinical trials [2, 3]. However, due to difficulties with administration of weekend doses, many centres either administer the same dose on a 5-day schedule (5–0-0), or a 5-day schedule followed by a weekend break followed by an additional 2 days (5–2-2) [4]. There has been no formal randomized clinical trial comparing the efficacy and tolerability of the alternative azacitidine doses, and the assumption has been that they are equivalent [5]. However, there are several important pharmacologic points that may challenge this assumption.

The active form of azacitidine binds both RNA and DNA, exerting its cytotoxic effect via interference with RNA transcription and DNA methyltransferase I activity in actively proliferating cells [1, 6]. In studies of azacitidine pharmacokinetics, the drug was undetectable in daily pretreatment blood samples, suggesting a rapid elimination and no accumulation [1]. Therefore, as the drug is only active in proliferating cells and does not accumulate, shorter durations of therapy within each cycle are less likely to have the drug encounter all malignant clones in their S-phase [1, 5]. This would conceptually argue for the increased efficacy of longer duration of treatment per cycle [5]. However, this argument does not discount interrupted courses of therapy such as 5–2-2. Since the benefit of azacitidine has most definitively been demonstrated in RCT using the 7–0-0 schedule, it becomes important to collect efficacy data on the alternative dosing schedules in order to ensure they are at least as equally effective as 7–0-0 [7]. The objective of the current systematic review is to evaluate the efficacy and tolerability of the 5–0-0, 5–2-2, and 7–0-0 azacitidine dosing regimens in MDS patients.

## Methods

The primary outcome was objective response rate (ORR) calculated as the combination of complete response (CR), partial response (PR), and hematological improvement (HI) as per the IWG 2006 criteria [8]. Due to the heterogeneity of the reporting of outcome data, the ORR was determined to be the outcome that could be extracted from the greatest number of articles and abstracts describing azacitidine treatment. Due to the inability to separate the treatment outcomes of AML patients that were included in retrieved studies of azacitidine therapy in MDS, these patients were included in the analysis of ORR.

### Search strategy

A systematic literature search was conducted in November 2014 and updated in October 2015 using the OVID interface and included MEDLINE, EMBASE, and Cochrane Central Register of Controlled Trials (Central) databases. The full methodology is described below with no additional review protocol or registration. No language restrictions were applied.

A sensitive search strategy was based on combination of subject headings and text-words using alternative spellings and word endings, such as but not limited to the search terms ‘AZA’, ‘azacitidine’, ‘azacytidine’, ‘vidaza’, ‘ladakamycin’, ‘myelodysplastic syndrome’, ‘myelodysplasia’, and ‘MDS’. Modifications to the search strategy were made for each database using appropriate thesaurus terms and fields. The Medline search strategy is indicated in Additional file 1. Articles were evaluated for inclusion based on the title and abstract. If an abstract was not available, an attempt was made to retrieve the full article for evaluation. Any articles retrieved with the search that included AML and CMML patients were included in the subsequent analysis if they met inclusion criteria.

### Assessment of study quality and data extraction

Articles meeting inclusion criteria were retrieved for full data extraction. The inclusion criteria were as follows: randomized clinical trials, observational prospective, and observational retrospective studies evaluating the clinical response of patients with myelodysplastic syndrome to azacitidine. Studies were excluded if they were phase I clinical trials, review articles, case series, or abstracts that were subsequently published in full form. Studies assessing AML and CMML patients retrieved with the search strategy were included in the analysis. Relevant data from included articles was extracted using a data collection form, and encompassed the disease characteristics of patients included in selected studies, vidaza dosing regimens used, and outcome variables. The primary outcome was objective response rate (ORR) calculated as the combination of complete response (CR), partial response (PR), and hematological improvement (HI) as per the International Working Group (IWG) criteria. In those publications where the ORR was directly reported as is defined by the IWG criteria, this ORR was recorded. In those publications which did not directly report the ORR but did report CR, PR, and/or HI as defined by the IWG, the ORR was calculated as the sum of available data. If the ORR could not be calculated from an abstract and/or article, then this publication was not included in the data analysis. Articles that reported objective response based on the IWG 2000 criteria were included in the analysis, with the justification based on the overall similarity in objective response using the two criteria as is shown in Additional file 2: Table S3. Articles that included only AML patients had objective response defined as CR + CRi + PR + HI, where HI referred to patients who did not attain the response criteria for CR/CRi or PR. Articles that reported on MDS and AML patients where the objective response could not be separated based on disease type were included in the analysis. If the outcome results reported in a publication could not be attributed to a particular dosing regimen of azacitidine, an attempt was made to contact the corresponding author in order to obtain this data. The quality of RCT, including any possible degree of bias in the study, was assessed according to the criteria proposed by Jadad et al. [9] Non-randomized observational studies were assessed with respect to attrition bias and reporting bias using the Cochrane Bias Assessment Tool [10].

### Statistical analysis

A meta-analysis of effect sizes of the articles meeting inclusion criteria was planned but could not be performed as there were insufficient RCT directly comparing the efficacy of various azacitidine dosing regimens. A pooled proportion analysis using a random effects model was conducted as previously described [11, 12]. The primary outcomes of interest were objective response rate and complete response as per IWG [8]. A z-test was used to assess for differences between effects, with a *p*-value <0.05 considered statistically significant. A sensitivity analysis was done evaluating the pooled proportion of ORR in subgroups of patients retrieved with the search strategy.

## Results

### Results of the search strategy from the systematic review

**Fig. 1:**
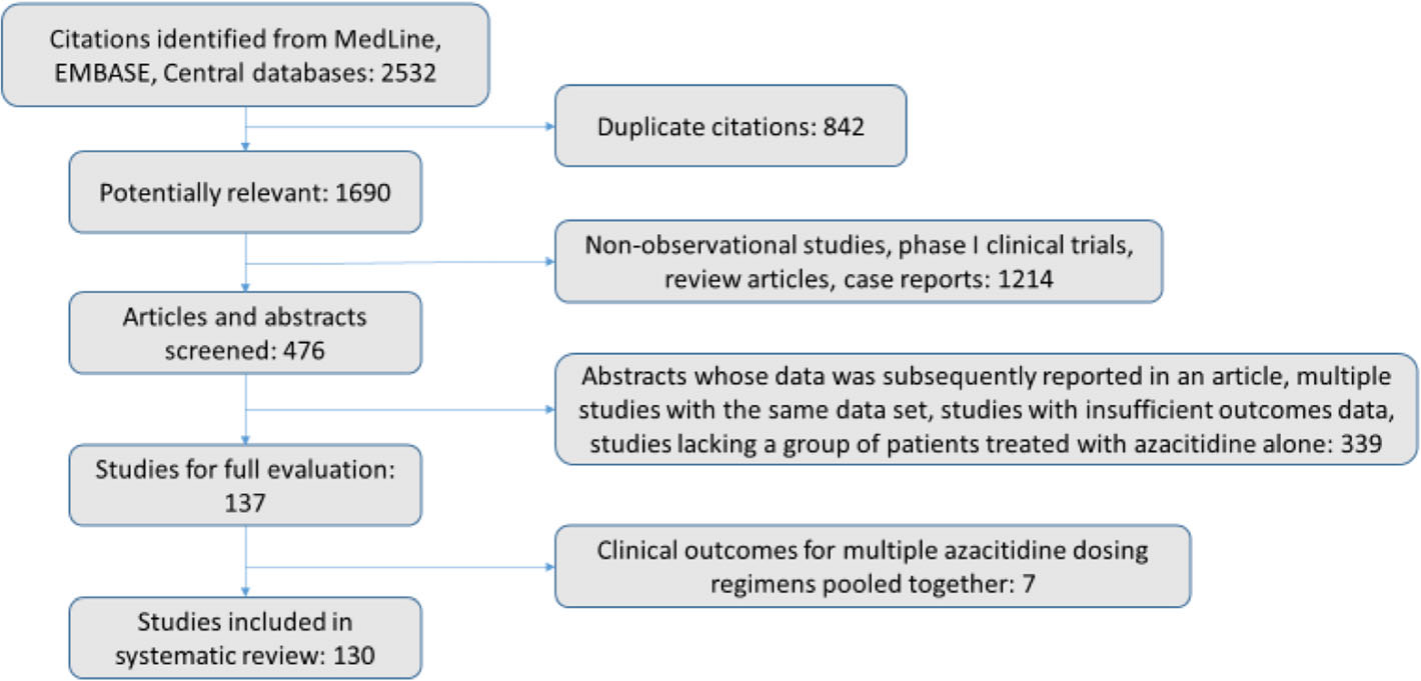
Flow diagram for the systematic review. The screening strategy resulted in the inclusion of only abstracts and articles for which objective response rate (ORR) as defined by the IWG 2006 criteria was either reported or could be calculated from the reported data for each particular dosing strategy. Seven studies that met all screening criteria were excluded from the final analysis because they reported ORR that was a pooled outcome for several different dosing regimens, and the raw outcome data for each particular dosing regimen could not be attained from the authors

**Fig. 2:**
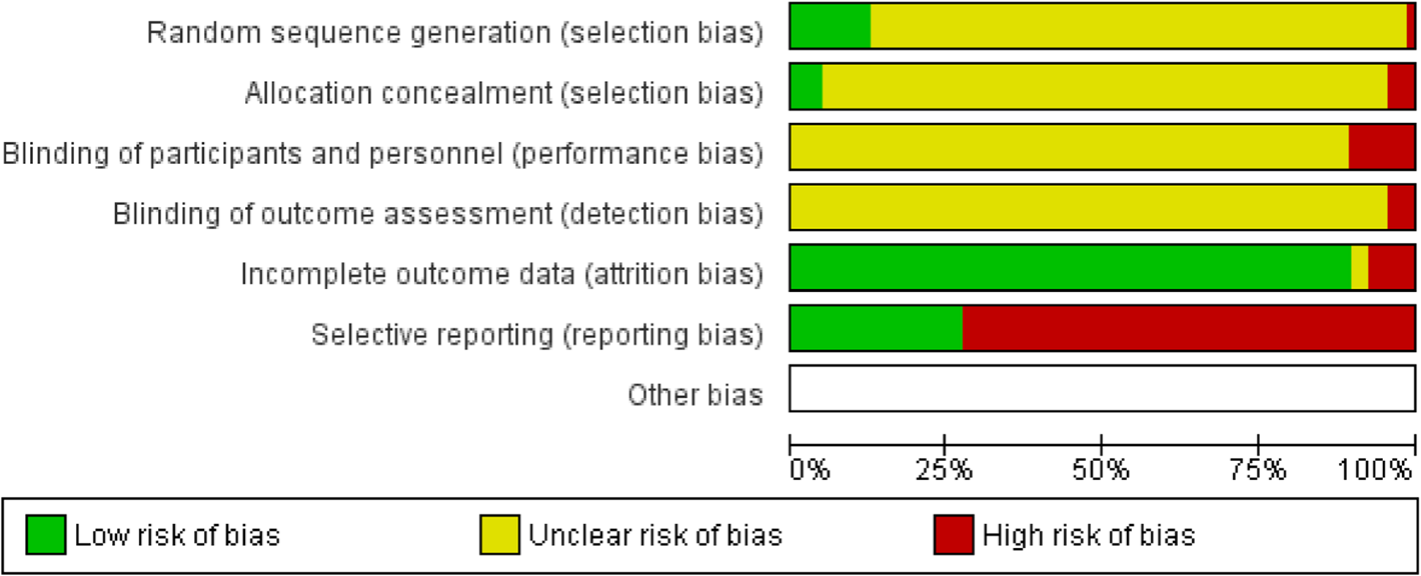
Risk of bias graph showing review authors’ judgements about each risk of bias item presented as percentages across all included studies. Every publication included in the systematic review was assessed for its risk of bias based on the reporting of data. Randomized clinical trials had the lowest risk of bias. The large amount of unclear risk of selection, performance, and detection bias reflects the relatively large number of non-randomized observational studies in the systematic review. The relatively high risk of reporting bias is a reflection of data acquired from conference abstracts that were judged to have a higher risk of selective reporting than full literature articles

The search strategy from all databases identified 1690 articles and abstracts after duplicates were removed, from which 47 articles and 90 abstracts met inclusion criteria for full study evaluation (Fig. 1). Of the 47 articles, there were 6 that did not report outcomes corresponding to individual azacitidine dosing regimens thereby excluding these articles from the final analysis. One article was excluded because the dosing regimen did not correspond to any of 5–0-0, 5–2-2, or 7–0-0, and another two articles were excluded because ORR could not be calculated. The remaining 38 articles along with the 90 abstracts were included in the pooled proportions analysis. As there were no randomized controlled trials directly comparing alternative dosing schedules of azacitidine, a meta-analysis of effects could not be performed. For most domains, studies had an unclear or a high risk of bias (Fig. 2). References to studies not cited in the article text but included in the data analysis are shown in Additional file 3.

### Characteristics of included studies

Of the 128 articles and abstracts meeting inclusion criteria, there were a total of 3 articles detailing randomized controlled trials (RCT) with one of the three articles summarizing data from three previous CALGB clinical trials (Table 1) [13]. Two of the RCTs evaluated the 7–0-0 regimen and one of the RCTs evaluated the 5–0-0 and 5–2-2 regimens, with all RCTs comparing azacitidine to conventional care that includes one or more of best supportive care, therapy with Ara-C, or intensive chemotherapy. The remainder of the articles were observational studies with either prospective (11/38) or retrospective (24/38) design.

**Table 1:** Characteristics of full studies reported in literature articles included in the systematic review

Study ID	Design	N	Inclusion criteria	Schedule	Cycles	Comparator	Concomitant therapy
Fenaux et al. [3]	RCT^	179	adults > = 18 with FAB diagnosis high risk MDS	7–0-0	9	CCR*: BSC, Ara-C, intensive chemo	none
Silverman et al. [13]	RCT	309	those used for the 3 clinical trials	7–0-0	3	BSC	none
Lyons et al. [14]	RCT	151	age > =18 with FAB‡ diagnosis RA/RARS/RAEB/RAEB-T/CMML and life expectancy >7 months	5–0-0, 5–2-2, 5–2-5	6	none	none
Xicoy et al. [15]	OR^^	107	MDS patients older than 75 treated with AZA	5–0-0, 5–2-2, 7–0-0	8	none	none
Garcia-Delgadoa et al. [16]	OR	200	age > =18 with either WHO-defined MDS or confirmed diagnosis of de novo/secondary AML with 20–30% blasts according to WHO who received at least 1 cycle of AZA	5–0-0, 7–0-0, 5–2-2	6, 8, 8	none	none
Sadashiv et al. [24]	OP^*	15	newly diagnosed AML who were deemed poor candidates for induction therapy and had an ECOG ≤2	5–0-0	5	none	none
Minoia et al. [25]	OR	18	therapy related MDS and AML not eligible for intensive chemotherapy	7–0-0	6	none	none
Drummond et al. [26]	OP	30	CMML-2 or CMML-1 patient with symptomatic marrow failure or proliferative disease	5–2-2	7	none	none
Fianchi et al. [27]	OR	31	consecutive patients receiving 5-aza	7–0-0	4	none	none
Ballya et al. [28]	OR	62	patients with diagnosis of MDS, CMML, or AML treated with AZA	7–0-0	8	none	none
Breccia et al. [29]	OP	38	WHO-diagnosed MDS patients treated with AZA† outside clinical trial	5–2-2	5	none	none
Breccia et al. [30]	OP	60	unselected WHO†‖-diagnosed MDS/CMML	5–2-2	6	none	none
Douvali et al. [31]	OR	42	intermediate-2/high risk MDS patients with normal hepatic function, ECOG 0–2	7–0-0	5.5	none	G-CSF
Duong et al. [32]	OR	84	patients with diagnosis of MDS or AML previously treated with chemotherapy having received at least 1 dose of AZA	7–0-0	4.5	none	none
Ettou et al. [33]	OR	169	consecutive patients treated with AZA between 2005 and 2011	7–0-0	6	none	none
Fianchi et al. [34]	OR	50	patients with therapy-related myeloproliferative neoplasms	7–0-0	4	none	ESA†* (8%), AML IC†** (12%)
Fil et al. [35]	OP	32	age > =18 with IPSS†† low/int-1 MDS and one or more of: (i) symptomatic anemia requiring RBC transfusion-supportive therapy, previously unresponsive to EPO or not expected to respond to EPO, (ii) thrombocytopenia requiring platelet transfusion, (iii) > 3 months ANC** less than 1.5	5–0-0	8	none	none
Gryna et al. [36]	OR	48	MDS patients, previous cytokine therapy allowed, ECOG <2 included	7–0-0	6	none	none
Itzykson et al. [37]	OR	86	MDS and AML patients treated with AZA	7–0-0	6	none	none
Itzykson et al. [38]	OR	282	IPSS Int-2/hi MDS patients as well as AML patients with blasts <30%	7–0-0, 5–0-0	6	none	none
O’Reilly et al. [23]	OR	47	elderly AML patients	5–0-0	5	none	none
Lee et al. [39]	OR	75	MDS patients treated with AZA	7–0-0	5	Decitabine	none
Lee et al. [40]	OR	203	patients needed to have an International Prognostic Scoring System (IPSS) lower risk score (IPSS low or intermediate-1) with significant cytopenia, or a higher risk score (IPSS intermediate-2 or high)	7–0-0	5	Decitabine	none
Al-Ali et al. [41]	OP	40	patients >18, life expectancy >2 months, with WHO-defined AML	5–0-0	3	none	none
Martin et al. [42]	OP	22	age > =18 with diagnosis of MDS based on FAB criteria, ECOG status <=2, adequate renal and hepatic function, no chemotherapy withing 4 weeks of enrollment	5–0-0	4.5	none	none
Moon et al. [43]	OR	129	MDS patients treated with Azacitidine	7–0-0	3	none	G-CSF, EPO
Muller-Thomas et al. [44]	OR	32	MDS and sAML patients treated with Azacitidine	7–0-0	4	none	RA†***, VA in 2 patients
Muller-Thomas et al. [45]	OP	100	MDS patients treated with Azacitidine	7–0-0	4	none	none
O’Reilly et al. [46]	OR	32	consecutive treatment-naïve patients treated with AZA between 2006 and 2012	5–0-0	9	none	none
Ozbalak et al. [47]	OR	25	MDS, AML, and CMML patients not eligible for chemotherapy treated with azacitidine	7–0-0	8	none	none
Papoutselis et al. [48]	OR	87	late-stage MDS, ECOG 0–2	7–0-0	6	BSC	G-CSF
Pierdomenico et al. [49]	OR	50	consecutive patients treated with AZA between 2005 and 2011	5–0-0	7.5	none	none
Tobiasson et al. [50]	OP	30	age greater than 18 with IPSS low/int-1 or mixed MDS/myeloproliferative disorder, CMML less than 10% marrow blasts or RARS	5–0-0	6	none	none
Diamantopoulos et al. [51]	OR	44	higher risk MDS or AML with 20–30% bone marrow blasts	7–0-0	5	none	none
Passweg et al. [24]	OP	45	elderly or frail patients with AML not eligible for intensive chemotherapy	5–0-0	4	none	none
van der Helm et al. [25]	OR	55	newly diagnosed AML receiving upfront treatment with 5-aza	7–0-0	6	none	none
van der Helm et al. [26]	OR	26	newly diagnosed AML	7–0-0	6	none	none

Note. Studies reported in abstracts were not included in this table. Refer also to Additional file 3

^RCT: randomized controlled trial, ^^OR: objective restrospective, ^*OP: objective prospective *CCR: conventional care regimen, BSC: best supportive care, **ANC: absolute neutrophil count, ***BM: bone marrow, †AZA: azacitidine, ‡FAB: French-American-British classification, ††IPSS: International Prognostic Scoring System, †‖WHO: World Health Organization, †* Erythropoiesis stimulating agents, †** Intensive chemotherapy, †*** Retinoic acid, ††* Valproic acid

A summary of the patient characteristics of included studies is shown in Additional file 2: Table S1. There were a total of 7520 patients, with 5545 patients receiving the 7–0-0 regimen, 1207 receiving the 5–0-0 regimen, and 768 receiving the 5–2-2 regimen. The median age of all patients was 70. The median age of all patients reported in articles with the 5–0-0, 5–2-2, and 7–0-0 regimens was 66, 72, and 69, respectively. The mean number of cycles received by patients treated with the 5–0-0, 5–2-2, and 7–0-0 regimens was 6, 6.7, and 5.5, respectively. An r by c chi square test was done showing that it was statistically more likely for patients receiving the 7–0-0 treatment regimen to have IPSS high risk than it was for the other treatment groups χ^2^(2, *N* = 2050) = 13.33, *p* = 0.0013. Similarly, it was statistically more likely for patients receiving the 7–0-0 treatment regimen to have a diagnosis of AML χ^2^(2, *N* = 2760) = 121.4, *p* < 0.000001. For articles that reported ECOG values, those articles reporting on patients treated with the 5–0-0, 5–2-2, and 7–0-0 regimens had 80%, 75%, and 80% of their patients in an ECOG <= 1 group, respectively. An r by c chi square test was done showing that there was no statistically significant association between the proportion of patients with ECOG score ≤ 1 and treatment regimen χ^2^(2, *N* = 1825) = 0.681, *p* = 0.7114.

### Direct comparison of dosing regimens

The outcomes from the articles included in the systematic review are summarized in Additional file 2: Table S2. There was a small number of studies directly comparing the different azacitidine regimens. There were two studies directly comparing the 5–0-0 and 5–2-2 dosing regimens. One was a randomized controlled trial showing no statistically significant difference in ORR between the 5–0-0 and 5–2-2 dosing regimens [14]. The other was an observational retrospective study that also showed no difference in ORR between the two regimens [15]. There were also two studies directly comparing the 5–0-0 and 7–0-0 regimens, both of which are observational retrospective studies that showed no statistically significant difference in ORR between the two regimens [15, 16]. Due to methodological heterogeneity, we did not conduct a direct comparison between groups.

### Results of the pooled proportions analysis

The pooled proportion of ORR was 44.8% (95% CI 42.8% to 45.5%) for the 7–0-0 dosing regimen, 41.2% (95% CI 39.2% to 41.9%) for the 5–0-0 regimen, and 45.8% (95% CI 42.6% to 46.4%) for the 5–2-2 regimen. A sensitivity analysis was done evaluating the pooled proportion of ORR in subgroups of patients such as those strictly reported to have a diagnosis of MDS, to have higher risk disease based on the IPSS, and based on the type of study that was performed (observational prospective, observational retrospective, RCT). Results of the sensitivity analysis are shown in Table 2.

**Table 2:** Sensitivity analysis of objective response rate of azacitidine in MDS

	Objective Response Rate Random Effects Model	
	Pooled rate (%)	CI
All patients (N* = 7520)		
7–0-0 (*N* = 5545)	44.8	(42.8, 45.5)
5–0-0 (*N* = 1207)	41.2	(39.2, 41.9)
5–2-2 (*N* = 768)	45.8	(42.6, 46.4)
MDS patients only (*N* = 2966)		
7–0-0 (*N* = 2187)	45.9	(44.1, 46.7)
5–0-0 (*N* = 536)	39.9	(36.8, 40.5)
5–2-2 (*N* = 243)	50.6	(48.7, 51.3)
IPSS int-2/hi patients (*N* = 1180)		
7–0-0 (*N* = 926)	46.8	(44.9, 47.3)
5–0-0 (*N* = 112)	54.6	(53.8, 55.0)
5–2-2 (*N* = 142)	60.7	(59.0, 61.5)
Randomized Controlled Trials (*N* = 883)		
7–0-0 (*N* = 440)	43.5	(43.0, 43.7)
5–0-0 (*N* = 320)	38.0	(35.4, 38.2)
5–2-2 (*N* = 123)	48.2	(48.0, 48.6)
Prospective Observational Studies (*N* = 1131)		
7–0-0 (*N* = 401)	45.9	(44.2, 46.9)
5–0-0 (*N* = 481)	39.3	(35.8, 40.1)
5–2-2 (*N* = 249)	40.0	(34.0, 40.5)
Retrospective Observational Studies (*N* = 4930)		
7–0-0 (*N* = 3910)	46.8	(44.4, 47.5)
5–0-0 (*N* = 624)	46.2	(44.9, 47.0)
5–2-2 (*N* = 396)	49.8	(47.5, 50.6)

*N refers to the number of patients included in a study

## Discussion

This systematic review was intended to test the hypothesis of whether the more practically convenient 5–0-0 and 5–2-2 azacitidine dosing regimens used to treat MDS have at least equivalent efficacy to the approved 7–0-0 dosing regimen studied in randomized clinical trials. The number of studies directly comparing the alternative dosing regimens was small, and no study directly compared all three regimens to each other. In those studies where a comparison of alternative regimens was made, they were found to be equivalent in terms of the ORR. Unfortunately, methodological heterogeneity of studies prevented a meta-analysis of effects.

The choice of ORR as the primary outcome variable was made due to the heterogeneity of the reporting of outcome data in articles describing azacitidine therapy. The ORR was determined to be able to pool data from the greatest amount of published literature on the subject of azacitidine dosing. Due to the heterogeneity of the reporting of survival data and to the substantial number of articles and abstracts that did not report overall survival, this outcome could not be used in the pooled proportions analysis. Other outcome variables commonly reported in studies of azacitidine, including CR, PR, HI, and transfusion dependence were independently evaluated as potential primary outcome variables for the pooled proportions analysis, and none encompassed as many studies as the ORR. Only the reporting of CR was similar to that of the ORR, with far more heterogeneity of reporting noted for the other outcome variables. Similarly to ORR, the attainment of stable disease has been found to have a correlation with overall survival in MDS, and was considered for inclusion in the pooled proportion analysis [17]. However, stable disease as an outcome was reported in a total of 42% of articles and abstracts. The exclusion of all articles and abstracts not reporting on stable disease would result in a greater degree of bias affecting the interpretation of the outcomes.

The definition of ORR did undergo an update in 2006, resulting in differences in the way this value was calculated from patient data compared to preceding years [8, 18]. Although this is a limitation of choosing the ORR as an outcome variable, the majority (75%) of the ORR used in the pooled proportions analysis was determined using the IWG 2006 criteria. Furthermore, due to the significant similarities between the IWG 2000 and IWG 2006 definitions of ORR, the relatively small number of articles and abstracts included in this review that reported the ORR using the IWG 2000 definition is unlikely to significantly affect the pooled proportions analysis. Additional file 2: Table S3 compares the IWG 2000 and IWG 2006 criteria for ORR.

The inclusion of CMML and AML patients in this systematic review was required because it was not possible to separate the outcomes of these patients from the MDS patients in most studies. Excluding any study that reported on CMML or AML in addition to MDS would have resulted in a substantial reduction in the total number of studies and patients as is shown in the sensitivity analysis (Table 2). For studies that reported on AML patients included in the review, response outcomes were reported allowing for the determination of ORR [19, 20–22]. Two studies of AML patients retrieved with the search strategy that did not report on HI were excluded from the pooled proportion and sensitivity analyses because ORR could not be calculated.

A pooled proportions analysis of the different dosing regimens across both randomized and observational studies was performed. Understanding the inherent limitation of this analysis [11, 12], it was found that the 7–0-0, 5–2-2, and 5–0-0 regimens had pooled ORR of 44.8%, 45.8%, and 41.2%, respectively. Interestingly, the confidence intervals of the 7–0-0 and 5–0-0 regimens do not overlap in a random effects model of pooled proportions, suggesting the possibility that the 7–0-0 may have somewhat greater efficacy in terms of the ORR than the 5–0-0 regimen. This as an indirect comparison of pooled ORR, but lends support to the idea that total time of exposure to azacitidine does play a role in clinical efficacy [6]. The same outcome is noted for the indirect comparison of the ORR of the 5–2-2 regimen and 5–0-0 regimen, also suggesting the possibility that a longer exposure to azacitidine has clinical benefit. Indirect comparison of the 7–0-0 and 5–2-2 regimens yielded overlapping confidence intervals, suggestive of the equal efficacy of these regimens in terms of ORR. What seems to be consistent is that a total course of 7 days (with or without a weekend break) of treatment with azacitidine has a statistically significant higher pooled ORR than a 5-day course.

It is important to note that the pooled set of patients receiving the 7–0-0 treatment regimen had a greater proportion of patients with IPSS high risk score and a diagnosis of AML than the other two treatment regimens. This likely reflected the fact that the 7–0-0 regimen was studied in clinical trials and is the regimen receiving clinical approval. How this impacted the pooled ORR for this group of patients across all studies is not clear because the IPSS is a prognostic score predictive of survival in MDS, not objective response rate [23]. To determine whether the higher proportion of AML patients treated with the 7–0-0 regimen affected the outcome of the pooled proportion analysis, a sensitivity analysis was performed assessing the response of patients with a diagnosis of MDS only (studies assessing any patients with a diagnosis of AML or CMML were excluded). It yielded the same outcome in that the pooled ORR with the 7–0-0 and 5–2-2 regimens were higher than the pooled ORR with the 5–0-0 regimen. The slightly higher ORR of the 7–0-0 in relation to the 5–0-0 regimen in an indirect pooled proportional analysis was consistent in similar sensitivity analyses focusing on patients assessed in randomized clinical trials, and on patients assessed in prospective observational studies (Table 2). The clinical significance of this finding is uncertain, however, without a direct comparison of the different dosing regimens in a clinical trial.

An important limitation of the current systematic review is that due to a paucity of randomized controlled trials in directly comparing the alternative azacitidine dosing regimens, most of the articles and abstracts included in this systematic review refer to observational prospective and retrospective studies. With a lack of randomization, blinding, and allocation concealment in these studies, there is a substantial risk of selection, performance, and detection bias as summarized in Fig. 2 [10]. However, with the consistency of the finding that the pooled ORR for a total of 7 days of azacitidine exposure is higher that the pooled ORR for 5 days of exposure, a randomized clinical trial is required for direct comparison and a definitive answer. If a trial is not performed, a standardization of outcome data reporting in the literature would facilitate the update of the sort of analysis done in this study with the inclusion of stable disease and survival as outcomes.

## Conclusions

In summary, this systematic review of alternative azacitidine dosing regimens in MDS and AML patients has highlighted an important deficiency in the literature regarding outcome reporting. Based on a small number of studies directly comparing alternative dosing regimens, there is no difference in efficacy of the 7–0-0, 5–2-2, and 5–0-0 dosing regimens in attaining ORR. However, an indirect comparison of the dosing regimens in the form of a pooled proportions analysis encompassing all studies on the subject yielded a slightly higher ORR for a total of 7 days of exposure to azacitidine as compared to 5 days. A prospective randomized clinical trial directly comparing the three dosing regimens is required to definitively address this comparison. Furthermore, a standardization of the reporting of outcomes of azacitidine treatment would facilitate future indirect comparisons of dosing regimens if a randomized trial is not preformed.

## Additional files

Additional file 1: This file includes the Medline search strategy used. (DOCX 13 kb)

Additional file 2: This file includes **Tables S1**, **S2**, and **S3.** The former describes the available characteristics of all patients included in this study that were reported in literature articles (abstracts did not provide sufficient information regarding baseline patient characteristics). **Table S2.** describes available objective response rate and survival data from literature articles retrieved for this systematic review. **Table S3.** is a comparison of the IWG 2000 and IWG 2006 objective response criteria. (DOCX 31 kb)

Additional file 3: This file includes the supplementary reference list for the systematic review. These references include articles and abstracts retrieved for this systematic review that were not cited in the main manuscript, but that are included in the data analysis. (DOCX 20 kb)
