# Supplementary material for: Systematic review of azacitidine regimens in myelodysplastic syndrome and acute myeloid leukemia
Source: BMC Hematol. 2018 Jan 31;18:3. doi: 10.1186/s12878-017-0094-8 (PMC5793426; doi:10.1186/s12878-017-0094-8)
Supplement: Supplementary file 1 — This file includes the Medline search strategy used. (DOCX 13 kb) [file 12878_2017_94_MOESM1_ESM.docx]

| **Supplementary Table 1. Characteristics of patients included in the systematic review.** | | | | | | | | | | | | | | | | | | |
| --- | --- | --- | --- | --- | --- | --- | --- | --- | --- | --- | --- | --- | --- | --- | --- | --- | --- | --- |
| **Study ID** | **Aza* dosing schedule** | **Mean Age** | **Male (%)** | **ECOG** | | **FAB†** | | | | **WHO‡** | | | | | | | **IPSS†† Score** | |
|  |  |  |  | **0/<=1 (%)** | **>=2**  **(%)** | **RA (%)** | **RAEB**  **(%)** | **CMML(%)** | **Unclassified (%)** | **del5q(%)** | **RA(%)** | **RCMD(%)** | **RAEB(%)** | **CMML(%)** | **AML**  **(%)** | **unclassified(%)** | **low/int-1(%)** | **int-2/hi**  **(%)** |
| Fenaux et al. [3] | 7-0-0 | 69 | 74 | 92 | 8 | 0 | 92 | 3 | 5 | 0 | 0 | 0 | 63 | 6 | 30 | 1 | 3 | 97 |
| Silverman et al. [13] | 7-0-0 | 65 | 65 | 58 | 21 | 0 | 100 | 0 | 0 | NR | NR | NR | NR | NR | 52 | NR | NR | NR |
|  | 7-0-0 | 66 | 67 | 74 | 7 | 16 | 50 | 20 | 14 |  |  |  |  |  | 37 |  |  |  |
|  | 7-0-0 | 69 | 72 | 70 | 9 | 22 | 56 | 12 | 10 |  |  |  |  |  | 27 |  |  |  |
| Lyons et al. [14] | 5-0-0 | 73 | 56 | 84 | 16 | 58 | 30 | 12 | 0 | NR | NR | NR | NR | NR | NR | NR | NR | NR |
|  | 5-2-2 | 76 | 73 | 84 | 16 | 55 | 35 | 10 | 0 |  |  |  |  |  |  |  |  |  |
| Xicoy et al. [15] | 5-0-0 | 78 | 65 | NR | NR | 30 | 57 | 7 | 6 | NR | NR** | NR | NR | NR | NR | NR | 45 | 55 |
|  | 7-0-0 |  |  |  |  |  |  |  |  |  |  |  |  |  |  |  |  |  |
|  | 5-2-2 |  |  |  |  |  |  |  |  |  |  |  |  |  |  |  |  |  |
| Garcia-Delgadoa et al. [16] | 5-0-0  5-2-2  7-0-0 | 70  69  70 | 70  64  67 | 73  78  59 | 22  14  29 | NR | NR | NR | NR | 2  5  0 | 12  7  22 | 20  25  24 | 45  43  31 | 0  0  0 | 1  3  8 | 20  17  15 | 69  70  55 | 31  30  45 |
| Sadashiv et al. [20] | 5-0-0 | 74 | 60 | 80 | 20 | NR | NR | NR | NR | 0 | 0 | 0 | 0 | 0 | 100 | 0 | NR | NR |
| Minoia et al. [21] | 7-0-0 | 65 | 39 | 56 | 44 | NR | NR | NR | NR | 0 | 0 | 6 | 67 | 0 | 22 | 5 | NR | NR |
| Drummond et al. [22] | 5-2-2 | 70 | 67 | NR | NR | NR | NR | NR | NR | 0 | 0 | 0 | 0 | 100 | 0 | 0 | NR | NR |
| Fianchi et el. [23] | 7-0-0 | 69 | 74 | 84 | 16 | 0 | 0 | 100 | 0 | 0 | 0 | 0 | 0 | 0 | 100 | 0 | 42 | 58 |
| Ballya et al. [24] | 7-0-0 | 71 | 61 | NR | NR | NR | NR | NR | NR | 3 | 0 | 11 | 50 | 6 | 30 | 0 | 10 | 90 |
| Breccia et al. [25] | 5-2-2 | 70.2 | 84 | 79 | 21 | NR | NR | NR | NR | 0 | 0 | 18 | 71 | 11 | 0 | 0 | 0 | 100 |
| Breccia et al. [26] | 5-2-2 | 69 | 73 | NR | NR | NR | NR | NR | NR | 0 | 0 | 13 | 80 | 7 | 0 | 0 | 0 | 100 |
| Douvali et al. [27] | 7-0-0 | 74 | 74 | NR | NR | NR | NR | NR | NR | 0 | 0 | 7 | 50 | 19 | 14 | 10 | 0 | 100 |
| Duong et al. [28] | 7-0-0 | 65 | 56 | 73 | 27 | NR | NR | NR | NR | 0 | 7 | 32 | 50 | 1 | 9 | 1 | 20 | 80 |
| Ettou et al. [29] | 7-0-0 | NR | NR | NR | NR | NR | NR | NR | NR | 5 | 37 | 10 | 28 | 0 | 20 | 0 | NR | NR |
| Fianchi et al. [30] | 7-0-0 | 66 | 56 | 92 | 8 | NR | NR | NR | NR | 0 | 0 | 24 | 44 | 0 | 32 | 0 | NR | NR |
| Fil et al. [31] | 5-0-0 | 71 | 69 | NR | NR | NR | NR | NR | NR | 0 | 66 | 19 | 15 | 0 | 0 | 0 | 100 | 0 |
| Gryna et al. [32] | 7-0-0 | 71 | 63 | NR | NR | 38 | 44 | 18 | 0 | NR | NR | NR | NR | NR | NR | NR | 46 | 54 |
| Itzykson et al. [33] | 7-0-0 | 71 | 65 | NR | NR | NR | NR | NR | NR | 0 | 0 | 0 | 73 | 0 | 27 | 0 | 13 | 87 |
| Itzykson et al. [34] | 7-0-0  5-0-0 | 71 | 61 | 77 | 23 | NR | NR | NR | NR | 0 | 4 | 0 | 73 | 0 | 23 | 0 | 0 | 100 |
| O’Reilly et al. [35] | 5-0-0 | 70 | 55 | NR | NR | NR | NR | NR | NR | 0 | 0 | 0 | 0 | 0 | 100 | 0 | NR | NR |
| Lee et al. [36] | 7-0-0 | 58 | 67 | 73 | 27 | NR | NR | NR | NR | 0 | 20 | 25 | 53 | 1 | 0 | 1 | 55 | 45 |
| Lee et al. [37] | 7-0-0 | 64 | 65 | NR | NR | NR | NR | NR | NR | 1 | 12 | 28 | 52 | 0 | 0 | 7 | 60 | 40 |
| Al-Ali et al. [38] | 5-0-0 | 72 | 48 | NR | NR | NR | NR | NR | NR | 0 | 0 | 0 | 0 | 0 | 100 | 0 | NR | NR |
| Martin et al. [39] | 5-0-0 | 69.5 | 59 | 86 | 9 | 27 | 64 | 9 | 0 | 0 | 14 | 14 | 54 | 9 | 9 | 0 | 40 | 60 |
| Moon et al. [40] | 7-0-0 | 64 | 65 | 91 | 9 | NR | NR | NR | NR | 1 | 26 | 27 | 43 | 0 | 0 | 13 | 67 | 33 |
| Muller-Thomas et al. [41] | 7-0-0 | 71.3 | 69 | NR | NR | NR | NR | NR | NR | 0 | 7 | 15 | 50 | 0 | 25 | 3 | 32 | 68 |
| Muller-Thomas et al. [42] | 7-0-0 | 71 | NR | NR | NR | NR | NR | NR | NR | 0 | 0 | 6 | 94 | 0 | 0 | 0 | 0 | 100 |
| O’Reilly et al. [43] | 5-0-0 | 73 | 72 | NR | NR | NR | NR | NR | NR | 0 | 6 | 6 | 60 | 25 | 0 | 3 | NR | NR |
| Ozbalak et al. [44] | 7-0-0 | 70 | 80 | 72 | 28 | NR | NR | NR | NR | 0 | 0 | 12 | 36 | 20 | 32 | 0 | 59 | 41 |
| Papoutselis et al. [45] | 7-0-0 | 73.5 | 72 | NR | NR | NR | NR | NR | NR | 0 | 0 | 2 | 41 | 0 | 30 | 27 | 0 | 100 |
| Pierdomenico et al. [46] | 5-0-0 | 66.5 | 58 | NR | NR | NR | NR | NR | NR | 0 | 2 | 4 | 42 | 16 | 36 | 0 | 14 | 86 |
| Tobiasson et al. [47] | 5-0-0 | 69 | 70 | NR | NR | NR | NR | NR | NR | 3 | 10 | 60 | 10 | 0 | 0 | 17 | 100 | 0 |
| Diamantopoulos et al. [48] | 7-0-0 | 73 | 68 | NR | NR | NR | NR | NR | NR | 0 | 2 | 7 | 64 | 10 | 17 | 0 | 7 | 93 |
| Passweg et al. [49] | 5-0-0 | 74 | 60 | NR | NR | NR | NR | NR | NR | 0 | 0 | 0 | 0 | 0 | 100 | 0 | NR | NR |
| van der Helm et al. [50] | 7-0-0 | 73 | 75 | NR | NR | NR | NR | NR | NR | 0 | 0 | 0 | 0 | 0 | 100 | 0 | NR | NR |
| van der Helm et al. [51] | 7-0-0 | 70 | 65 | 81 | 19 | NR | NR | NR | NR | 0 | 0 | 0 | 0 | 0 | 100 | 0 | NR | NR |

*Aza: Azacitidine

**NR: not reported.

†FAB: French-American-British classification system

‡WHO: World Health Organization

††IPSS: International Prognostic Scoring System

Supplementary Table 2. Outcomes including Objective Response Rate (ORR), Overall Survival (OS), and Treatment Modification from articles and abstracts included in the systematic review

| **Study ID** | **Azacitidine dosing schedule** | **Objective response rate (%)** | **Overall survival (OS)** | **Percentage of patients requiring Azacitidine dose**  **reduction or discontinuation due to adverse events (%)** |
| --- | --- | --- | --- | --- |
| Fenaux et al. [3] | 7-0-0 | 78 | Median OS: 24.5 months | 5 |
| Silverman et al. [13] | 7-0-0 | 45 | Median OS: 19.3 months | NR |
| Lyons et al. [14] | 5-0-0  5-2-2 | 56  45 | NR | 34  63 |
| Xicoy et al. [15] | 5-0-0  7-0-0  5-2-2 | 40  33  37 | 2-yr OS: 34%, with no significant difference between the treatment groups | 37  -  - |
| Garcia-Delgadoa et al. [16] | 5-0-0  7-0-0  5-2-2 | 29  41  45 | Median OS: 13.2 monts  Median OS: 14.9 months  Median OS: 19.1 months | 14  14  29 |
| Sadashiv et al. [20] | 5-0-0 | 47 | NR | 27 |
| Minoia et al. [21] | 7-0-0 | 36 | Median OS: 9.6 months | 17 |
| Drummond et al. [22] | 5-2-2 | 20 | NR | NR |
| Fianchi et al. [23] | 7-0-0 | 51 | NR | NR |
| Ballya et al. [24] | 7-0-0 | 45 | Median OS: 15.6 months | NR |
| Breccia et al. [25] | 5-2-2 | 78 | Median OS: 16.4 months | NR |
| Breccia et al. [26] | 5-2-2 | 63* | Median OS: 21 months (low risk FPSS)  15 months (intermediate risk FPSS)  11 months (high risk FPSS) | NR |
| Douvali et al. [27] | 7-0-0 | 38 | Median OS: 10.4 months | 29 |
| Duong et al. [28] | 7-0-0 | 37 | Median OS: 14.5 months | NR |
| Ettou et al. [29] | 7-0-0 | 52 | NR | NR |
| Fianchi et al. [30] | 7-0-0 | 33 | Median OS: 21 months | NR |
| Fil et al. [31] | 5-0-0 | 57 | Median OS: 28.5 month | NR |
| Gryna et al. [32] | 7-0-0 | 32 | NR | 22 |
| Itzykson et al. [33] | 7-0-0 | 39 | Median OS: 15.3-17.5 months | NR |
| Itzykson et al. [34] | 7-0-0  5-0-0 | 44  41 | Median OS: 14.3 months  Median OS: 10.3 months | 7 |
| O’Reilly et al. [35] | 5-0-0 | 32 | NR | NR |
| Lee et al. [36] | 7-0-0 | 48 | OS (2-year): 42.1%  IPSS Int-1 MDS: median OS 19.9 months  IPSS Int-2/Hi MDS: median OS 16.8 months | 10 |
| Lee et al. [37] | 7-0-0 | 34 | Median OS: 23.2 months | NR |
| Al-Ali et al. [38] | 5-0-0 | 30 | Median OS: 3 months | NR |
| Martin et al. [39] | 5-0-0 | 28 | Median OS: 14.8 months | 50 |
| Moon et al. [40] | 7-0-0 | 65 | Median OS: 20 months | NR |
| Muller-Thomas et al. [41] | 7-0-0 | 6 | Median OS: 15 months | 18 |
| Muller-Thomas et al. [42] | 7-0-0 | 28 | Median OS: 12.6 months | NR |
| O’Reilly et al. [43] | 5-0-0 | 47 | Median OS: 20 months | NR** |
| Ozbalak et al. [44] | 7-0-0 | 40 | OS 28% with median follow-up 13 months | NR |
| Papoutselis et al. [45] | 7-0-0 | 41 | Median OS: 11.8 months  OS (1-year): 46%, OS (2-year): 23% | NR |
| Pierdomenico et al. [46] | 5-0-0 | 48 | Median OS: 19.3 months. 40% of patients alive at median follow-up of 14.3 months | 4 |
| Tobiasson et al. [47] | 5-0-0 | 10 | At 30 month follow-up: OS 60% | 60 |
| Diamantopoulos et al. [48] | 7-0-0 | 34 | NR | 73 |
| Passweg et al. [49] | 5-0-0 | 34 | Median OS: 6 months | NR |
| Van der Helm et al. [50] | 7-0-0 | 42 | Median OS: 12.3 months | NR |
| van der Helm et al. [51] | 7-0-0 | 42 | NR | NR |

*ORR is 63% in the group with MDS and second simultaneous cancer, and 69% in the group with secondary MDS or AML

**NR: not reported

Supplementary Table 3. Comparison of the objective response rate (ORR) as defined by the IWG 2000 and IWG 2006 criteria

| **ORR (IWG 2006) = CR + PR + HI** | **ORR (IWG 2000) = CR + PR + HI** |
| --- | --- |
| **CR***   - < 5% marrow blasts without evidence of dysplasia - normalization of peripheral blood counts   Hemoglobin > 11 g/dL  Neutrophil count > 1 x 10^9^ /L  Platelet count > 100 x 10^9^ /L    **Marrow CR**   - Bone marrow < 5% myeloblasts and decrease > 50% over pretreatment - Peripheral blood: if HI responses, they are noted in addition to marrow CR | **CR**   - < 5% marrow myeloblasts with normal maturation of all cell lines - Normalization of peripheral blood counts   Hemoglobin > 11 g/dL  Neutrophil count > 1.5 x 10^9^ /L  Platelet count > 100 x 10^9^ /L |
| **PR**   - Marrow blasts decrease by 50% or more compared to pretreatment levels but still > 5% - normalization of peripheral blood counts   Hemoglobin > 11 g/dL  Neutrophil count > 1 x 10^9^ /L  Platelet count > 100 x 10^9^ /L | **PR**   - Marrow blasts decrease by 50% or more compared to pretreatment levels, or less advanced MDS FAB classification than pretreatment - normalization of peripheral blood counts   Hemoglobin > 11 g/dL  Neutrophil count > 1.5 x 10^9^ /L  Platelet count > 100 x 10^9^ /L |
| **Cytogenetic response**   - Complete: disappearance of chromosomal abnormality without appearance of new one - Partial: 50% or more reduction of chromosomal abnormality | **Cytogenetic response**   - Major: no detectable cytogenetic abnormality - Minor: 50% or more reduction of abnormal metaphase |
| **Hematologic improvement (HI)**  **HI = HI-E + HI-P + HI-N**  **HI-E**:   - for patients with pretreatment Hb < 11 g/dL, greater than 1.5 g/dL increase in hemoglobin; for RBC transfusion-dependent patients, reduction of at least 4 RBC transfusions over 8 week period   **HI-P**:   - for patients with pretreatment platelet count > 20 x 10^9^ /L, an absolute increase of 30 x 10^9^ /L or more - increase from < 20 x 10^9^ /L to > 20 x 10^9^ /L and by at least 100%   **HI-N**:   - at least 100% increase and an absolute increase of > 0.5 x 10^9^ /L | **Hematologic improvement (HI)**  **HI = HI-E + HI-P + HI-N**  **HI-E**:   - Major: for patients with pretreatment Hb < 11 g/dL, greater than 2 g/dL increase in hemoglobin; for RBC transfusion-dependent patients, transfusion independence - Minor: for patients with pretreatment Hb < 11 g/dL, 1-2 g/dL increase in hemoglobin; for RBC transfusion-dependent patients, 50% decrease in transfusion requirements   **HI-P**:   - Major: for patients with pretreatment platelet count < 100 x 10^9^ /L, an absolute increase of 30 x 10^9^ /L or more; for platelet transfusion-dependent patient, stabilization of platelet counts and transfusion independence - Minor: for patients with pretreatment platelet count < 100 x 10^9^ /L, a 50% or more increase in platelet count with a net increase greater than 10 x 10^9^ /L but less than 30 x 10^9^ /L   **HI-N**:   - Major: for absolute neutrophil count less than 1500/mm^3^ before therapy, at least 100% increase, or an absolute increase of more than 500/mm^3^, whichever is greater - Minor: for absolute neutrophil count less than 1500/mm^3^ before therapy, at least 100% increase but absolute increase less than 500/mm^3^ |

*CR: complete response, PR: partial response, HI: hematologic improvement, HI-E: hematologic improvement-erythroid, HI-P: hematologic improvement-platelet, HI-N: hematologic improvement-neutrophil
