# Supplementary material for: Systematic review of azacitidine regimens in myelodysplastic syndrome and acute myeloid leukemia
Source: BMC Hematol. 2018 Jan 31;18:3. doi: 10.1186/s12878-017-0094-8 (PMC5793426; doi:10.1186/s12878-017-0094-8)
Supplement: Supplementary file 2 — This file includes Tables S1, S2, and S3. The former describes the available characteristics of all patients included in this study that were reported in literature articles (abstracts did not provide sufficient information regarding baseline patient characteristics). Table S2. describes available objective response rate and survival data from literature articles retrieved for this systematic review. Table S3. is a comparison of the IWG 2000 and IWG 2006 objective response criteria. (DOCX 31 kb) [file 12878_2017_94_MOESM2_ESM.docx]

**Supplementary Reference List**

1. Improta S, Gagliardi A, Villa MR, *et al*. Azacitidine therapy in high-risk myelodysplastic syndromes (MDS): A single centre experience. Leukemia Research. 2013; 37[suppl.1]: S152 – S152.

2. Kim YK, Kim HJ, Song JE, *et al*. Treatment outcome of 5'-azacitidine for myelodysplastic syndrome. In: 12th International Symposium on Myelodysplastic Syndromes; 2013, May 8-11; Berlin, Germany. Leukemia Research. 2013; 37[suppl.1]: S1-S117, P-193.

4. Drummond MW, Pocock C, Boissinot M, *et al*. A multi-centre phase 2 study of azacitidine in chronic myelomonocytic leukaemia. Leukemia. 2014; 28: 1570-1572.

5. Bally C, Ades L, Renneville A, *et al*. Prognostic value of TP53 gene mutations in higher-risk MDS treated with azacitidine (AZA). Leukemia Research. 2013; 38(7): 751-755.

6. Annereau M, Desmaris R, Micol JB, *et al*. Results of treatment with 5-azacytidine in patients treated for myelodysplastic syndrome or secondary acute myeloid leukemia associated with a concomitant active cancer. In: 2014 ASCO Annual Meeting; 2014, May 30 – June 3; Chicago, IL. Journal of Clinical Oncology. 2014; 32[suppl]: abs e18029.

7. Gardin C, Thépot S, Beyne-Rauzy O, et al. Results of a phase II trial of azacitidine (AZA) with or without epoetin beta (EPO) in lower-risk MDS. In: 2012 ASCO Annual Meeting; 2012, June 1 – 5; Chicago, IL. Journal of Clinical Oncology. 2012; 30[suppl]: abs 6523.

8. Pappa V, Fragoulakis V, Maniadakis N, *et al*. The overall response rate of azacitidine in patients with intermidiate-2 and high risk myelodysplastic syndromes: A retrospective chart review study from Greece. Value in Health. 2014; 17(3): A223-A224.

9. Garcia-Manero G, Huang X, Cabrero M, *et al*. A bayesian phase II randomized trial of azacitidine versus azacitidine + vorinostat in patients with newly diagnosed AML or high-risk MDS with poor performance status, organ dysfunction, or other comorbidities. In: 56th Annual ASH Meeting and Exposition; 2014, Dec 6-9; San Francisco, CA; Blood. 2014; 124(21): 3277-3277.

10. Lee YG, Kim I, Yoon SS, *et al*. Head-to-head comparison of 5-azacitidine versus decitabine for the treatment of myelodysplastic syndrome. In: 54th Annual ASH Meeting and Exposition; 2012, Dec 8-11; Atlanta, GA; Blood. 2012; 120(21): 2843-2843.

11. Vidal V, Ginet C, Karsenti JM, *et al*. BCL2L10 quantification is a predictive factor of response to azacitidine in myelodysplastic syndromes (MDS) and acute myeloid leukemia (AML). In: 56th Annual ASH Meeting and Exposition; 2014, Dec 6-9; San Francisco, CA; Blood. 2014; 124(21): 3261-3261.

12. Nazha A, Kantarjian HM, Cortes JE, *et al*. Outcome of patients with myelodysplastic syndrome (MDS) with bone marrow (BM) blasts between 10 to 30% treated with hypomethylating agents versus intensive chemotherapy. In: 54th Annual Meeting of the American Society of Hematology; 2012, Dec 8-11; Atlanta, GA; Blood. 2012; 120(21) (no pagination).

13. Ivanoff S, Lemasle E, Gruson B, *et al*. Azacitidine in relapsed and refractory AML: Efficacy for patients relapsing as MDS post AML. In: 54th Annual ASH Meeting and Exposition; 2012, Dec 8-11; Atlanta, GA; Blood. 2012; 120(21) (no pagination).

14. Nam S-H, Lim T, Kim BS. Outcome of 5-Azacitidine and decitabine treatment in elderly patients with myelodysplastic syndrome. In: 54th Annual ASH Meeting and Exposition; 2012, Dec 8-11; Atlanta, GA; Blood. 2012; 120(21) (no pagination).

15. Freyrie A, Reda G, Vincenti D, *et al*. 5-Azacitidine therapy in patients with myelodysplastic syndromes, chronic myelomonocytic leukemia and acute myeloid leukemia: A single institution experience. In: 54th Annual ASH Meeting and Exposition; 2012, Dec 8-11; Atlanta, GA; Blood. 2012; 120(21) (no pagination).

16. Guolo F, Minetto P, Clavio M, *et al*. Azacytidine therapy in previously untreated elderly AML patients: Comparison with conventional chemotherapy in a matched-paired analysis. In: 45th Congress of the Italian Society of Hematology; 2015, October 4-7; Florence, Italy; Haematologica. 2015; 100[suppl.3]: P218.

17. Falantes J, Deben G, Robles VM, *et al*. Azacitidine in older patients with acute myeloid leukemia (AML) and adverse karyotype. Subanalisis from the alma study. In: 56th Annual ASH Meeting and Exposition; 2014, Dec 6-9; San Francisco, CA; Blood. 2014; 124(21): 945.

18. Sekeres MA, Othus M, List AF, *et al*. A Randomized Phase II Study of Azacitidine Combined with Lenalidomide or with Vorinostat Vs. Azacitidine Monotherapy in Higher-Risk Myelodysplastic syndromes (MDS) and Chronic Myelomonocytic Leukemia (CMML): North American Intergroup Study SWOG S1117. In: 56th Annual ASH Meeting and Exposition; 2014, Dec 6-9; San Francisco, CA; Blood. 2014; 124(21): LBA-5.

19. Kyo T, Kyo K, Toishikawa K, *et al*.Treatment of AML with myelodysplasia related changes(AML-MRC) By sequential therapy of azacitidine (AZA) and intensive chemotherapy. In: 56th Annual ASH Meeting and Exposition; 2014, Dec 6-9; San Francisco, CA; Blood. 2014; 124(21):5288-5288.

20. Desoutter J, Gay J, Berthon C, *et al*. Molecular prognostic factors in Acute Myeloid Leukemia (AML) patients receiving first line therapy with azacytidine (AZA). In: 56th Annual ASH Meeting and Exposition; 2014, Dec 6-9; San Francisco, CA; Blood. 2014; 124(21): 482-482.

21. Quinto AM, De ME, Castelli M, *et al*. Infections in patients with myelodysplastic syndrome/acute myeloid leukemia treated with azacitidine: Report from a single center. In: 56th Annual ASH Meeting and Exposition; 2014, Dec 6-9; San Francisco, CA; Blood. 2014; 124(21): 5622-5622.

22. Freyrie A, Guidotti F, Reda G, Binda F, Cortelezzi A. Single institution experience of 5-azacitidine therapy in patients with myelodysplastic syndromes and acute myeloid leukemia. In: 17th Congress of the European Hematology Association; 2012, Jun 14-17; Amsterdam, Netherlands; Haematologica. 2012; 97[suppl.1]: 587-587.

23. Boehrer S, Beyne-Rauzy O, Prebet T, *et al*. Interim Results of A Randomized Phase II Trial of Azacitidine (AZA) +/- Epo In Lower Risk Myelodysplastic Syndrome (MDS) Resistant to An Erythropoietic Stimulating Agent (ESA) Alone. In: 52nd Annual ASH Meeting and Exposition; 2010, Dec 6-9; San Diego, CA; Blood. 2010; 116(21): 1880-1880.

24. Castelli M, Binotto G, Pavanello F, Ermani M, Zambello R, Semenzato G. Impact of comorbidity on treatment with 5-azacitidine and outcome in 39 patients with myelodysplastic syndrome: A single center population analysis. In: 17th Congress of the European Hematology Association; 2012, Jun 14-17; Amsterdam, Netherlands; Haematologica. 2012; 97[suppl.1]: 586-586.

25. Bergua JM, Arcos-Carmona M, Prieto-Fernandez J, *et al*. Azacytidine 75mg/m2 x5 day in myelodisplastic syndromes and acute myeloid leukemia refractory/relapsed patients: Results from a single centre. In: 17th Congress of the European Hematology Association; 2012, Jun 14-17; Amsterdam, Netherlands; Haematologica. 2012; 97[suppl.1]: 582-582.

26. Kotsianidis I, Douvali E, Papoutselis M, *et al*. Safety and efficacy of 5-azacytidine in mds patients with moderate renal impairment: A single center experience. In: 17th Congress of the European Hematology Association; 2012, Jun 14-17; Amsterdam, Netherlands; Haematologica. 2012; 97[suppl.1]: 580-580.

27. Fiallo D, Luzardo H, Suarez A, *et al*. Experience of a 5-day schedule of azacitidine in a singlecentre acute myeloid leukemia population. In: 17th Congress of the European Hematology Association; 2012, Jun 14-17; Amsterdam, Netherlands; Haematologica. 2012; 97[suppl.1]: 506-506.

28. Wolfromm A, Dreyfus F, Vey N, *et al*. Treatment of advanced CMML by azacitidine (AZA) in a compassionate program. The GFM experience in 38 patients (pts). In: 52nd Annual ASH Meeting and Exposition; 2010, Dec 6-9; San Diego, CA; Blood. 2010; 116(21): 4023-4023.

29. Gardin C, Thepot S, Beyne-Rauzy O, *et al*. Results of a phase ii trial of azacitidine (AZA)+/-epoetin beta (EPO) in lower risk MDS. In: 17th Congress of the European Hematology Association; 2012, Jun 14-17; Amsterdam, Netherlands; Haematologica. 2012; 97[suppl.1]: 361-361.

30. Kartsios H, Loukidis K, Papadopoulos V, *et al*. The combination of Azacitidine (AZA) and recombinant erythropoietin (rEPO) can induce rapidly hematological response in intermediate-2 and high-risk MDS (including RAEB-t). In: 52nd Annual ASH Meeting and Exposition; 2010, Dec 6-9; San Diego, CA; Blood. 2010; 116(21): 2914-2914.

31. Drummond W, Boissinot M, Cauchy P, *et al*. Cmml201: A phase 2 trial of azacitidine in chronic myelomonocytic leukaemia. In: 17th Congress of the European Hematology Association; 2012, Jun 14-17; Amsterdam, Netherlands; Haematologica. 2012; 97[suppl.1]: 358-358.

32. Field T, Perkins J, Nishihori T, *et al*. Prospective trial of pre-transplant 5-azacitidine on hematopoietic cell transplantation outcomes for myelodysplastic syndrome and CMML. In: 52nd Annual ASH Meeting and Exposition; 2010, Dec 6-9; San Diego, CA; Blood. 2010; 116(21): 1333-1333.

33. Ricco A, Carluccio P, Russo RA, *et al*. Long-term outcome of higher-risk MDS patients treated with azacitidine: Single centre experience. In: XIII Congress of the Italian Society of Experimental Hematology; 2014, October 15-17; Rimini, Italy; Haematologica. 2014; 99[suppl.2]: S78-S78.

34. Iuliano F, Perricelli A, Iuliano E, *et al*. Assisted administration of subcutaneous 5-azacytidine to the patient's home is safe and cost-effective. In: 19th Congress of the European Society of Hematology Association; 2014, June 12-15; Milan, Italy; Haematologica. 2014; 99[suppl.1]: 622-622.

35. Abbas KS, Li A, Rivero GA, Lulla PD, Mulchandani A, Yasin Z, Yellapragada SV (2011) Outcome of myelodysplastic syndrome in veterans treated with 5-azacitidine and decitabine. In: 53rd Annual Meeting of the American Society of Hematology; 2011, Dec 10-13; San Diego, CA; Blood 118(21) (no pagination).

36. Navarro PG, Delgado RG, Garcia AB, Munoz JA. Clinical experience with azacitidine in Chronic Myelomonocytic Leukemia (CMML) in Spain. In: 53rd Annual Meeting of the American Society of Hematology; 2011, Dec 10-13; San Diego, CA. Blood. 2011; 118(21) (no pagination).

37. Chou WC, Yeh SP, Hsiao LT, *et al*. A phase 4, open-label, single-arm study to evaluate the efficacy, safety, and pharmacokinetics of subcutaneous azacitidine in adult taiwanese subjects with higher-risk myelodysplastic syndromes. In: 19th Congress of the European Society of Hematology Association; 2014, June 12-15; Milan, Italy; Haematologica. 2014; 99[suppl.1]: 612-612.

38. Sebert M, Lainey E, Lepelley P, *et al*. Expression and function of the P-glycoprotein (P-gp) in Myelodysplastic syndromes (MDS) treated with azacytidine (AZA). In: 53rd Annual Meeting of the American Society of Hematology; 2011, Dec 10-13; San Diego, CA; Blood. 2011; 118(21) (no pagination).

39. De Astis E, Guolo F, Clavio M, *et al*. Azacytidine therapy in untreated AML patients: Retrospective multicentre regional experience. In: 19th Congress of the European Society of Hematology Association; 2014, June 12-15; Milan, Italy; Haematologica. 2014; 99[suppl.1]: 577-577.

40. Papadopoulos V, Papageorgiou V, Karagianni A, *et al*. Combined use of azacitidine (AZA) & recombinant human erythropoietin (RHUEPO) induces rapid haematological response without affecting response rate in higher risk MDS: Results of a retrospective study. In: 18th Congress of the European Society of Hematology Association; 2013, June 13-16; Stockholm, Sweden; Haematologica. 2013; 98[suppl.1]: 583-583.

41. De Gusmao B, Campeny A, Perez-Persona E, *et al*. Monitoring of the cytogenetic alterations in patients with acute myeloid leukemia/myelodysplastic syndrome treated with azacitidine. In: 18th Congress of the European Society of Hematology Association; 2013, June 13-16; Stockholm, Sweden; Haematologica. 2013; 98[suppl.1]: 582-582.

42. Min Y-H, Yoon S, Hwang DY, *et al*. Circulating exosomal microRNA-21 as a potential biomarker that may predict response to azacitidine treatment in myelodysplastic syndromes. In: 53rd Annual Meeting of the American Society of Hematology; 2011, Dec 10-13; San Diego, CA; Blood. 2011; 118(21) (no pagination).

43. Capodanno I, Avanzini P, Merli F. 5-azacitidine in patients with myelodysplasia and acute myeloid leukemia: A single centre experience. In: 16th Congress of the European Society of Hematology Association; 2011, June 9-12; London, England; Haematologica. 2011; 96[suppl.2]: 519-519.

44. Sanna A, Gozzini A, Gioia D, *et al*. Comorbidities influence prognosis in MDS high-risk patients treated with 5-azacitidine. In: 16th Congress of the European Society of Hematology Association; 2011, June 9-12; London, England; Haematologica. 2011; 96[suppl.2]: 444-444.

45. Pierdomenico F, Parreira A, Almeida A. Efficacy and tolerability of 5-day azacytidine dose intensified regimen in higher risk MDS. In: 16th Congress of the European Society of Hematology Association; 2011, June 9-12; London, England; Haematologica. 2011; 96[suppl.2]: 349-349.

46. Passweg J, Pabst T, Blum S, *et al*. Azacytidine for acute myeloid leukemia in elderly or frail patients: A phase ii study (SAKK 30/07). In: 16th Congress of the European Society of Hematology Association; 2011, June 9-12; London, England; Haematologica. 2011; 96[suppl.2]: 256-256.

47. Pappa V, Vasilatou D, Papageorgiou S, *et al*. Treatment of intermediate and high risk myelodysplastic syndromes(MDS) with azacytidine. The hellenic experience. In: 16th Congress of the European Society of Hematology Association; 2011, June 9-12; London, England; Haematologica. 2011; 96[suppl.2]: 112-112.

48. O'Reilly M, McHale C, Almazmi A, *et al*. A 5-day outpatient regimen of 5-azacitidine is well-tolerated and effective for high-risk myelodysplastic syndrome and acute myeloid leukemia patients unsuitable for aggressive chemotherapy. In: 16th Congress of the European Society of Hematology Association; 2011, June 9-12; London, England; Haematologica. 2011; 96[suppl.2]: 110-110.

49. Rivellini F, Danise P, Califano C, *et al*. Clinical response to AZA in PTS with MDS/AML: A single center experience. In: 43rd Congress of the Italian Society of Hematology; 2011, October 16-19; Napoli, Italy; Haematologica. 2011; 96: 164-164.

50. Criscuolo M, Santini V, Fianchi L, *et al*. Assessement of response to 5-azacytidine in therapy related myeloid neoplasms. In: 43rd Congress of the Italian Society of Hematology; 2011, Oct 16-19; Napoli, Italy; Haematologica. 2011; 96: 162-162.

51. Breccia M, Cannella L, Mancini M, *et al*. 5-azacitidine feasibility and safety in elderly myelodysplastic patients. In: 43rd Congress of the Italian Society of Hematology; 2011, Oct 16-19; Napoli, Italy; Haematologica. 2011; 96: 158-159.

52. Serio B, Pezzullo L, Fontana R, *et al*. Prolonged azacitidine treatment in the management of elderly patients with high risk myelodysplastic syndromes, chronic myelomonocytic leukemia and acute myeloid leukemia not eligible for standard intensive therapy. In: 44th Congress of the Italian Society of Hematology; 2013, Oct 20-23; Verona, Italy. Haematologica. 2013; 98: 201-201.

53. Clissa C, Finelli C, Follo MY, *et al*. Azacitidine in high and low risk myelodysplastic syndromes: Retrospective evaluation of 57 patients treated with 4 different therapeutic regimens. In: 44th Congress of the Italian Society of Hematology; 2013, Oct 20-23; Verona, Italy; Haematologica. 2013; 98: 200-200.

54. Lanza Cariccio MR, Iovino G, Fadda MR, *et al*. Azacitidine is safe and well tolerated in very elderly patients affected by myelodysplastic syndromes. In: 44th Congress of the Italian Society of Hematology; 2013, Oct 20-23; Verona, Italy. Haematologica. 2013; 98: 199-199.

55. Follo MY, Malagola M, Fili C, *et al*. Pi-plcbeta1 gene methylation and expression as a reliable and dynamic marker of clinical response to 5-azacytidine in patients with low-risk myelodysplastic syndromes. In: 44th Congress of the Italian Society of Hematology; 2013, Oct 20-23; Verona, Italy; Haematologica. 2013; 98: 196-196.

56. Buquicchio C, Ciuffreda L, Santeramo TM, *et al*. Predictive platelet recovery and quality of life in elderly acute myeloid leukemia patients treated with azacytidine: Results of a retrospective single centre analysis. In: 44th Congress of the Italian Society of Hematology; 2013, Oct 20-23; Verona, Italy; Haematologica. 2013; 98: 115-115.

57. Buquicchio C, Santoro M, Ciuffreda L, *et al*. Monitoring response to treatment and minimal residual disease (MRD) by flow cytometry in erderly patients with acute myeloid leukaemia treated with 5-azacitidine: A single center experience. In: 12th Congress of the Italian Society of Experimental Hematology; 2012, Oct 17-19; Roma, Italy; Haematologica. 2012; 97: S60-S61.

58. Fili C, Follo MY, Martinelli G, *et al*. Is PI-PLC 1 expression a reliable predictor of response in low risk MDS patients treated with low-dose azacitidine? Results of a prospective multicentric study. In: 12th Congress of the Italian Society of Experimental Hematology; 2012, Oct 17-19; Roma, Italy. Haematologica. 2012; 97: S36-S36.

59. Sanna A, Donnini I, Clissa C, *et al*. Influence of bone marrow fibrosis on response of int-2/high risk MDS patients to 5-azacitidine. In: 11th Congress of the Italian Society of Experimental Hematology; 2010, Oct 6-8; Turin, Italy; Leukemia Research. 2010; 35(S1): S126-S127.

60. Passweg J, Pabst T, Blum S, *et al*. Chalandon SAKK Leukemia Project Group. Azacytidine for acute myeloid leukaemia in elderly or frail patients: a phase II study (SAKK 30/07). In: Jahrestagung der Deutschen, Osterreichischen und Schweizerischen Gesellschaften fur Hamatologie und Onkologie; 2011, September 30 – October 4; Basel, Switzerland; Onkologie. 2011; 34: 299-299.

61. Sanna A, Gozzini A, Donnini I, *et al*. Influence of mild bone marrow fibrosis on response of int-2/high risk MDS patients to 5-azacitidine. In: 11th International Symposium on Myelodysplastic Syndromes; 2011, May 19-22; Edinburgh, Scotland; Leukemia Research. 2011; 35[suppl.1]: S126-S127.

62. Iuliano F, Iuliano E, Perricelli A, Pomillo A, Luci M, Cervo G. Assisted administration of subcutaneous 5-azacytidine to the patient's home. Significant reduction of indirect costs incurred by care recipients and unpaid caregivers together with a total adhesion to the treatment schedule and same safety. In: 55th ASH Annual Meeting and Exposition; 2013, December 7-10; New Orleans, LA; Blood. 2013; 122(21): 5587-5587.

63. Jonasova A, Cermak J, Cervinek L, *et al*. Czech MDS Group experience with azacitidine in treatment of high risk MDS and AML patients. In: 11th International Symposium on Myelodysplastic Syndromes; 2011, May 19-22; Edinburgh, Scotland; Leukemia Research. 2011; 35[suppl.1]: S68-S68.

64. Bergua J, Arcos Carmona MJ, Fernandez JP, *et al*. Azacytidine 75mg/m2 ×5 day in high-risk myelodysplastic syndromes and acute myeloid leukemia refractory/relapsed patients: Results from a single centre. In: 11th International Symposium on Myelodysplastic Syndromes; 2011, May 19-22; Edinburgh, Scotland; Leukemia Research. 2011; 35[suppl.1]: S63-S63.

65. Bories P. AML therapy: Is azacytidine an alternative to intensive chemotherapy in older acute myeloid leukemia? A retrospective study on 334 older AML patients. In: Acute Leukemias 14th: Biology and Treatment Strategies; 2013, Feb 24-27; Munich, Germany; Annals of Hematology. 2013; 92: S4-S4.

66. Khan C, Rossetti JM, Hilton C, *et al*. Azacitidine as induction therapy of elderly patients with acute myelogenous leukemia. In: 2012 ASCO Annual Meeting; 2012, June 1 – 5; Chicago, IL. Journal of Clinical Oncology. 2012; 30[suppl]: abs 6620.

67. Maurillo L, Buccisano F, Gaidano G, *et al*. Intensive chemotherapy does not prolong survival compared to 5-azacitidine in patients aged 70 years or more with untreated acute myeloid leukemia. In: 54th Annual ASH Meeting and Exposition; 2012, Dec 8-11; Atlanta, GA; Blood. 2012; 120(21): 3596-3596.

68. Benjamin HL, Rossetti JM, Lampkin AJ, *et al*. Azacitidine in the treatment of elderly patients with acute myelogenous leukemia. In: 51st Annual Meeting of the American Society of Hematology; 2009, Dec 5-8; New Orleans, LA; Blood. 2009; 114 (22) (no pagination).

69. Kaya A, Tekgunduz E, Iskender D, Batgi H, Bekdemir F, Altuntas F. Retrospective evaluation of azacitidine monotherapy in patients with myelodysplastic syndrome: A single center experience. Leukemia Research. 2015; 39: S133-134.

70. Lestang E, Ayari S, Chevallier P, et al. 5-azacytidine (AZA) compared to intensive chemotherapy in elderly acute myeloid leukemia (AML) patients: Results of a retrospective single centre matched analysis. In: 53rd Annual Meeting of the American Society of Hematology; 2011, Dec 10-13; San Diego, CA; Blood. 2011; 118 (21) (no pagination).

71. Ricco A, Sgherza N, Rossi AR, et al. Long-term outcome of higher-risk MDS patients treated with azacitidine: Single centre experience. Blood. 2013; 122(21): 5220-5220

72. Sanna AL. Comorbidities do not modify outcome and frequency of adverse events during aza, but influence survival [abstract]. In: 15^th^ Congress of the European Hematology Association; 2010, June 10-13; Barcelona, Spain. Haematologica. 2010; 95 [suppl.2]: 376, abs 0905.

73. Freyrie A, Reda G, Vincenti D, et al. Results of 5-azacytidine therapy in patients with myelodysplastic syndromes (MDS) treated with azacitidine (AZA). A collaborative study in 878 patients. In: ASH 2013 Annual Meeting; New Orleans, LA; Blood. 2013; 122(21): abs 389.

74. Sebert M, Komrokji RS, Sekeres MA, et al. Impact of cytogenetics and cytogenetic response on outcome in Myelodysplastic syndromes (MDS) treated with azacitidine (AZA). A collaborative stuy in 878 patients. In: ASH 2013 Annual Meeting; New Orleans, LA; Blood. 2013; 122(21): abs 389.

75. Greco R, Riva M, Ravano E, Molteni A, Morra E. Azacitidine in high-risk MDS patients. A ‘real-life’ experience from a single center. Leukemia Research. 2013; 37: S157-S157.

76. Dwilewicz-Trojaczek J, Drozd-Sokolowska J, Waszczuk-Gajda A, et al. Factors predictive for response to azacitidine treatment in MDS patients – A report by Polish Adult Leukemia Group. Leukemia Research. 2013; 37[suppl.1]: S153-S154.

77. Woods A, Naassan A, Swain G, Schattner A. Efficacy and feasibility of azacitidine therapy for high-grade MDS in a community cancer center setting. Leukemia Research. 2015; 39: S126 – S126.

78. Pappa VP, Papageorgiou S, Tsirigotis P, et al. Treatment of intermediate and high risk myelodysplastic syndrome patients with azacitidine. The Hellenic experience. Leukemia Research. 2009; 33[suppl.1]: S134-S135.

79. Santini V, Lunghi T, Gozzini A, et al. Monocentric evaluation of response and tolerability of subcutaneous azacitidine in elderly MDS patients with comorbidities. Leukemia Research. 2009; 33(S1): S127-S128.

80. Dargenio M, Reddiconto G, Valacca A, et al. Efficacy and toxicity of intravenous 5-azacitidine (5-Aza) as single agent in the treatment of myelodisplastic syndromes (MDS) and unfit elderly acute myeloid leukemia (AML): Preliminary data of single-center experience. In: 42nd Congress of the Italian Society of Hematology; 2009, Oct 18-21; Milano, Italy; Haematologica: 2009; 94 (pp 130).

81. Bakanay SM, Karakilic E, Civriz-Bozdag S, et al. 5-azacytidine treatment results in myelodysplastic syndrome [abstract]. In: American Society of Clinical Oncology (ASCO) 45th Annual Meeting; 2009, May 29 – June 2; Orlando FL. Journal of Clinical Oncology. 2009; 27[suppl.15]: e18003.

82. Thepot S, Itzykson R, Raffoux E, et al. Treatment of progression of myeloproliferative neoplasm to MDS/AML by azacytidine: A report on 44 patients. In: 14th Congress of the European Hematology Association; 2009, June 4-7; Berlin, Germany. Haematologica. 2009; 94[suppl.2]: 438, abs. 1086.

83. Sohn SK, Baek JH, Moon JH, et al. Treatment outcome according to Pretreatment risk group in MDS patients who were treated with azacitidine. In: 14th Congress of the European Hematology Association; 2009, June 4-7; Berlin, Germany. Haematologica 94[suppl.2]: 101, abs. 0255.

84. Jonasova A, Cermak J, Cervinek L, *et al*. Azacitidine treatment of high risk MDS and AML, analysis of long responding patients and prognostic factors of overall survival, Czech MDS group experience. Leukemia Research. 2015; 39: S53-S53.

85. Improta S, Della CP, Esposito M, *et al*. Iron chelation therapy improves haematological response in high-risk myelodysplastic patients treated with azacitidine. Leukemia Research. 2015; 39[suppl.1]: S52-S53.

86. Binotto G, Quinto AM, De March E, *et al*. Infections in patients with myelodysplastic syndrome/acute myeloid leukemia treated with azacitidine: Retrospective analysis from a single center. Leukemia Research. 2015; 39[suppl.1]: S47-S47.

87. Cabrera CC, Falantes JF, Gómez M, *et al*. Azacitidine alone or the combination with valproic acid as a bridge therapy for acute myeloid leukemia and higher-risk myelodysplastic syndromes before transplantation. Blood. 2013; 122(21): 2812-2812

88. Yiu RL. Interim analysis of the use of 5-azacytidine for treatment of myelodysplastic syndrome/acute myeloid leukaemia with chromosome 7 or complex cytogenetic abnormalities [abstract]. In: 15^th^ Congress of the European Hematology Association; 2010, June 10-13; Barcelona, Spain. Haematologica. 2010; 95[suppl.2]: 376, abs. 0324

89. Kyo T, Kyo K, Imanaka R, *et al*. Improvement of the efficacy of azacitidine at a fixed dosing schedule of 75mg/m2/day for 5 days, followed by 21 days drug-off in higher risk myelodysplastic syndromes. Blood. 2013; 122(21): 1571-1571.

90. Legoupil C, Sebert M, Braun T, *et al*. Impact of myelofibrosis (MF) in MDS treated with azacitidine (AZA). A single center study. Blood. 2013; 122(21): 1539-1539.

91. Hirai M, Nakane T, Inoue A, *et al*. A new strategy of MDS treatment with azacitidine (AZA): Induction with AZA combined with low dose ARAC followed by maintenance therapy with AZA. Blood. 2013; 122(21): 1536-1536.
